# Public interest trends in regenerative injections for knee osteoarthritis: A 10-year Google Trends analysis

**DOI:** 10.1097/MD.0000000000049930

**Published:** 2026-08-07

**Authors:** Hakan Uslu, Oğuzhan Çiçek, Osman Çiloğlu

**Affiliations:** a Department of Orthopedics and Traumatology, University of Health Sciences, Adana City Training and Research Hospital, Adana, Turkey.

**Keywords:** Google Trends, hyaluronic acid injection, joint injection, knee osteoarthritis, platelet-rich plasma, stem cell therapy

## Abstract

This study aimed to determine public interest in intra-articular injection treatments for knee osteoarthritis (KOA) using Google Trends data. Relative search volumes (RSVs) for platelet-rich plasma (PRP), hyaluronic acid (HA), and stem cell treatments used in KOA treatment were analyzed using Google Trends data collected over the past decade (2015–2025). The annual search volume changes for each treatment were assessed using polynomial regression analysis. A subgroup analysis was performed based on the changes caused by COVID-19. The seasonal search trends were evaluated using the autoregressive integrated moving average model. Over the last 10 years, interest in the use of intra-articular injections for the treatment of KOA has increased. This increase was most pronounced in PRP. Although HA had a lower RSV than the other treatments, its trend toward a moderate increase was observed. Finally, although stem cells were the most common treatment between 2015 and 2020, their use has decreased in recent years. An increasing RSV trend was observed for PRP and HA over the past decade (*R*^2^ = 0.869, β = 7.364, *P* < .001; *R*^2^ = 0.880, β = 2.670, *P* < .001, respectively). Stem cells were the most common treatment between 2015 and 2020. They exhibited a decreasing trend in 2020 and beyond (*R*^2^ = 0.103, β = −1.002, *P* = .355). Seasonal analyses revealed no statistically significant differences in the number of searches across seasons. A dramatic decrease in the number of searches occurred during the COVID-19 pandemic. This decline is expected to revert to an increasing trend by 2022.

## 1. Introduction

Osteoarthritis (OA) is the most common form of arthritis, affecting approximately 15% of the global population. The prevalence of this condition increases with age. It is more common in women than men.^[1]^ It is the seventh most common cause of physical disability after the age of 70.^[2]^ Conservative treatments such as medical treatments and weight loss are used in the mild and moderate stages of this chronic disease, which increases in prevalence with age.^[3]^ However, many patients are unable to lose weight due to the limited mobility associated with OA.^[4]^ Total knee arthroplasty is considered a priority for patients who do not respond to lifestyle and medical treatments or are in advanced stages of the disease.^[3]^ However, some patients may be reluctant to undergo surgery or may choose not to undergo surgery due to complications. While total knee arthroplasty has a high success rate in patients whose various treatment options have failed, it is not without complications.^[5]^ In the last decade, intra-articular injections have been frequently used in patients with OA owing to their minimally invasive nature. Although evidence is still needed for hyaluronic acid (HA), stem cells, and platelet-rich plasma (PRP), these treatment methods have gained popularity in recent years.^[6]^

Google Trends objectively measures public interest in a topic by extracting data from Google, the world’s most widely used search engine. This tool analyzes a portion of the 3 billion daily Google searches and provides data on the geographical and temporal distributions of search volumes for user-specified terms.^[7]^ Understanding public interests and needs can guide health care professionals. Recently, research using Google Trends data to understand public interest has increased in medical literature. Various studies have examined stem cell, PRP, and HA injection treatments in patients with knee osteoarthritis (KOA).^[8-10]^ These studies reported a growing trend in the number of treatments used. Studies following the publication of these studies are limited, and studies comparing these treatments with each other using trend analyses appear to be lacking in the literature.

In this study, we aimed primarily to determine the evolution of public interest in the use of intraknee injections of PRP, stem cells, and HA for treating KOA over the past 10 years. Our second hypothesis was to examine how public interest in these treatments changed in relation to seasonal variations and outbreaks such as COVID-19.

## 2. Materials and methods

The data for this study were obtained using the publicly accessible Google Trends tool. Therefore, ethics committee approval was not required for this study. Google is the most widely used search engine in the world. Google Trends is a platform that systematically records and analyzes searches. The Google Trends platform objectively demonstrates changes in search terms over time, starting in 2004, and the differences in public interest across geography. Although these results are presented, they can also provide detailed data by city and country. The Google Trends platform displays normalized search interest for a specific search term, which is based on time and geography, and uses a relative index between 0 and 100, called the relative search volume (RSV). This index is calculated by assuming that the highest search volume for the relevant term in the selected time period is 100 and calculating the search volume in other time periods proportionally.

This study covers global Google searches between January 1, 2015 and January 1, 2025. The search terms were determined with a focus on intra-articular injection treatments used for KOA. Terms such as “platelet-rich plasma (PRP),” “stem cell,” and “hyaluronic acid” were searched in English and combined with the phrase “for knee” to ensure direct relevance (e.g., “prp for knee”). This minimized bias could be due to the use of related treatments for other indications. Terms were selected by consensus based on the researchers’ clinical experience, preliminary analyses, and Google Trends simulations.

After data collection, monthly and annual RSV values were compared and analyzed across the groups. In this study, a subgroup analysis was conducted using the COVID-19 period, which we believe significantly altered people’s interest in health concepts worldwide. This analysis included approximately 1 year before the COVID-19 pandemic to investigate how trends have changed since the present day. Another analysis was conducted to determine the seasonal variation in the public interest. These analyses were performed considering that a large part of the world’s population lives in the Northern Hemisphere (winter from December–January–February, spring from March–April–May, summer from June–July–August, and autumn from September–October–November).

### 2.1. Statistical analysis

Data were obtained from Google Trends and analyzed using SPSS 27.0 (IBM, Armonk) and R version 4.4.2 (R Foundation for Statistical Computing). Annual trends in RSVs were evaluated using polynomial regression, and monthly trends were assessed using the Mann–Kendall test. Time series forecasting was performed using an autoregressive integrated moving average (ARIMA) model. Model adequacy was assessed through visual agreement between observed and fitted values.

## 3. Results

Our study compared the relative search numbers for regenerative therapies used for knee disease over the last 10 years (2015–2025). First, although stem cells were the most commonly studied term for knee diseases between 2015 and 2020, they have shown a decreasing trend in recent years. Polynomial regression analysis evaluating search trends over time revealed a negative trend but no statistically significant difference (β = −1.002, *P* = .355; Table 1). Monthly changes via the Mann–Kendall test revealed a decrease in searches for stem cells, particularly for knee OA, in recent years (Tau coefficient = −0.171, *P* = .005; Table 2).

**Table 1:** Polynomial regression of Google search trends for yearly intra-articular injection of knee osteoarthritis.

Treatment	*R* ^2^	β	*P* value	95% CI
Platelet rich plasma	0.869	7.364	<.001	5.20–9.52
Stem cell	0.103	−1.002	.355	−3.22 to 1.22
Hyaluronic acid	0.880	2.670	<.001	1.93–3.41

CI = confidence interval.

**Table 2:** Mann–Kendall trend test results for monthly Google search volumes of knee regenerative treatment methods (2015–2025).

Treatment	Tau coefficient	*Z* value	*P* value
Platelet rich plasma	+0.8607	+14.166	<.001
Stem cell	−0.1714	−2.807	.005
Hyaluronic acid	+0.7552	+12.289	<.001

An examination of the number of searches and public interest in PRP for knee diseases revealed an increasing trend over both months and years. The average annual increase was β = 7.364 (*P* < .001). Furthermore, an increasing trend was observed in the use of HA for the treatment of knee OA over the years and months. The annual trend analysis via polynomial regression analysis is shown in Table 1, and the results of the monthly trend analysis via the Mann––Kendall test are shown in Table 2.

**Figure 1.**
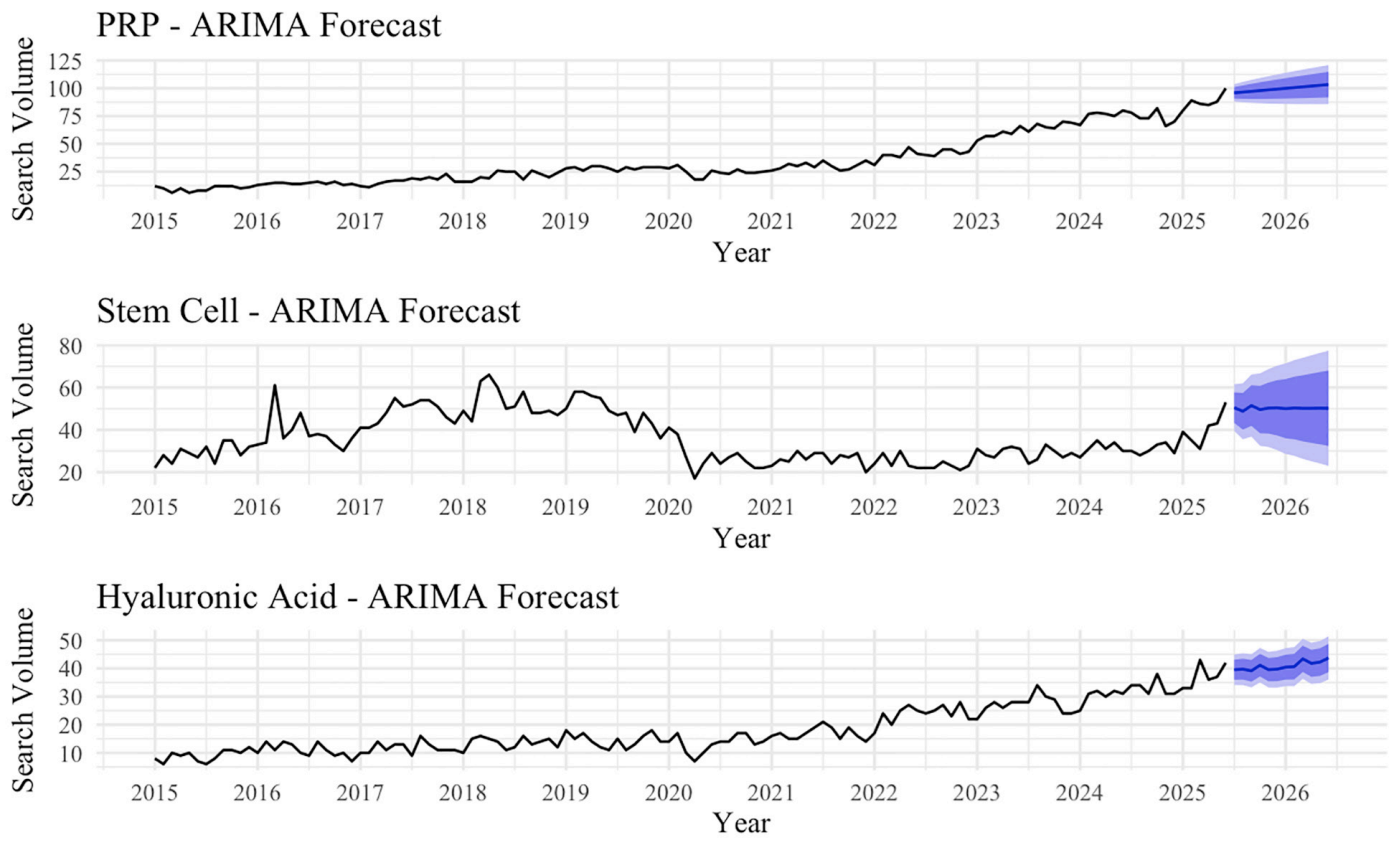
ARIMA-based forecasting of global search trends for PRP, stem cells, and hyaluronic acid in knee osteoarthritis. ARIMA = autoregressive integrated moving average, PRP = platelet-rich plasma.

**Figure 2.**
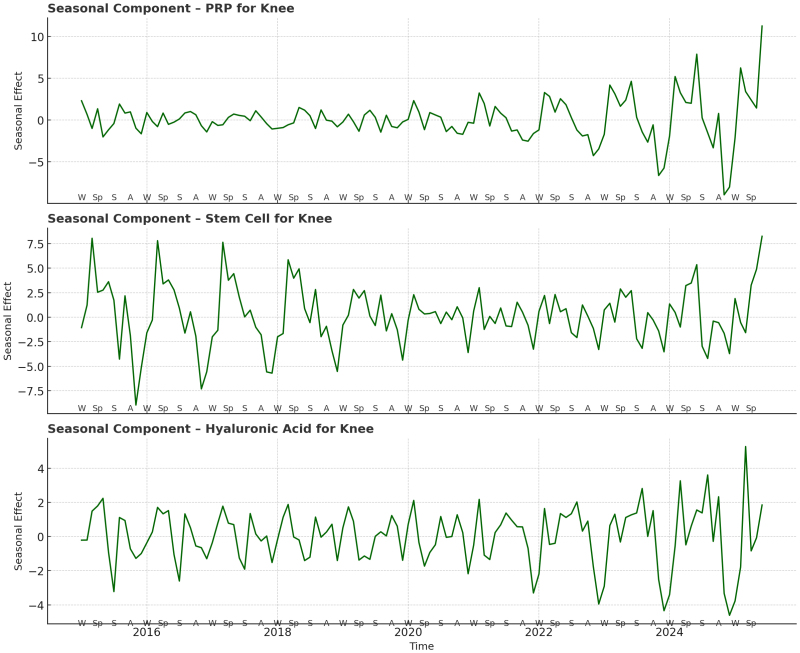
Seasonal trend analysis of intra-articular treatment searches over the last decade (2015–2025). PRP = platelet-rich plasma.

When treatment methods are evaluated via ARIMA forecasting, PRP will continue to increase in 2026, whereas HA will continue to increase moderately (Fig. 1). The decrease in stem cell searches in recent years is expected to continue until 2026. Subgroup analysis conducted during the COVID-19 pandemic revealed a significant decrease across all treatments, and this decline continued until January 2022. In the seasonal analyses (Fig. 2), the mean RSVs for PRP in winter, spring, summer, and autumn were 34.5 ± 23.3, 36.9 ± 25.6, 35.1 ± 24.1, and 33.4 ± 20.6, respectively, and no statistically significant difference was found between the seasons (*P* = .955). For stem cells, the values were 34.03 ± 9.3, 28.06 ± 13.9, 36.0 ± 12.0, and 34.6 ± 9.4, and for HA, the values were 17.2 ± 7.6, 19.1 ± 9.5, 18.4 ± 9.5, and 18.1 ± 7.9, respectively (*P* = .502 and *P* = .856, respectively).

**Figure 3.**
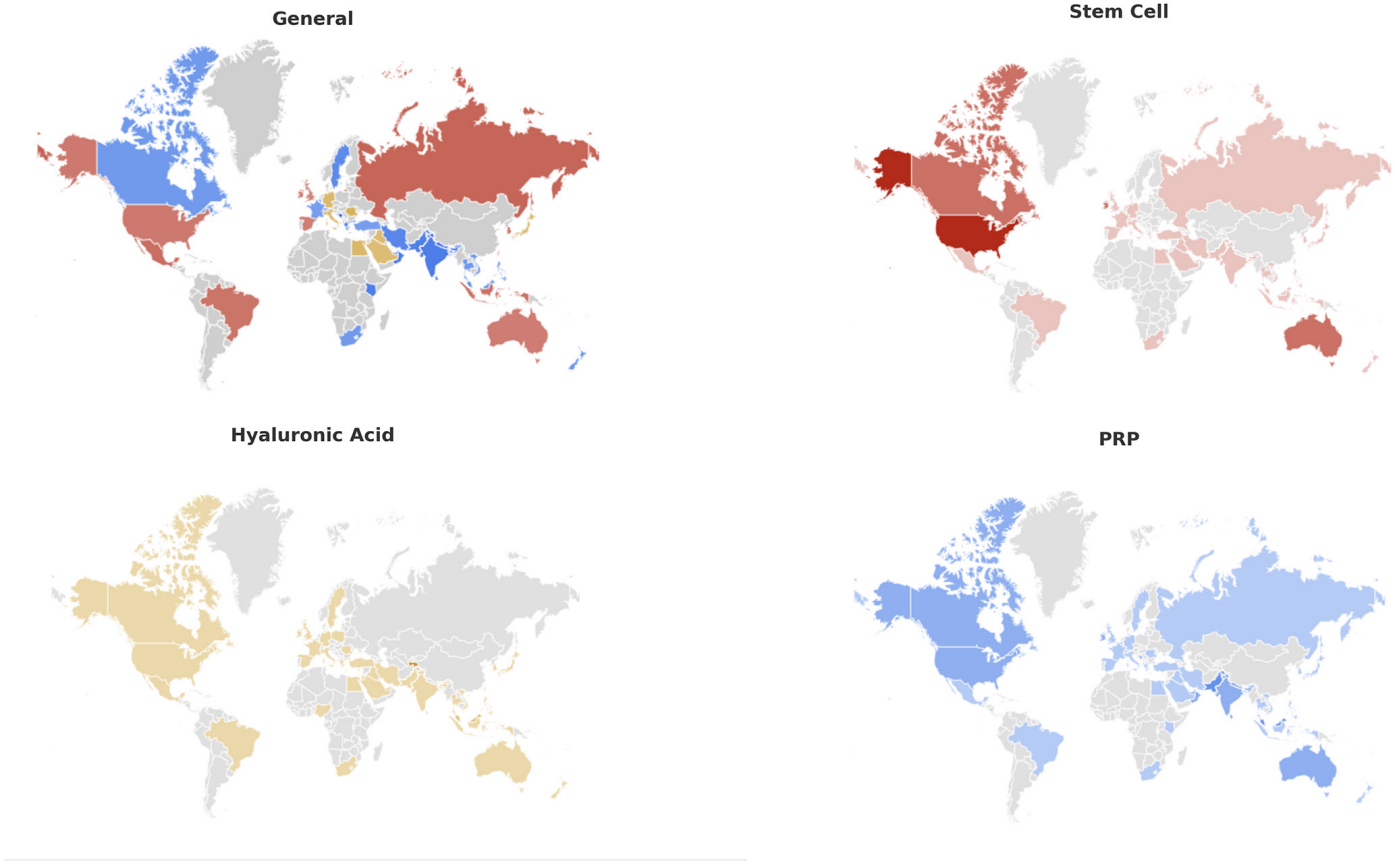
Global distribution of relative search interests by treatment type for knee osteoarthritis. PRP = platelet-rich plasma.

A global assessment of trends revealed that the Maldives have received the most searches for PRP in the last 10 years, relative to its population. The use of stem cells in the United States was explored, whereas the use of HA in Malaysia was explored. The top 5 countries with the highest global search results are listed in Table 3 and Figure 3.

**Table 3:** Top 5 countries by relative search volume for each selected regenerative knee therapy on the basis of Google Trends data.

PRP	Stem cell	Hyaluronic acid
Trinidad Tobago (100)	United States (100)	Malesia (100)
Sri Lanka (75)	Australia (50)	Cyprus (100)
Bahreyn (50)	Ireland (50)	United Arab Emirates (100)
Pakistan (50)	Canada (50)	Singapore (100)
Jamica (50)	United Arab Emirates (<1)	United States (<1)

PRP = platelet-rich plasma.

## 4. Discussion

This study evaluated the use of intra-articular knee injections to treat OA in recent years using Google Trends analysis. The main finding of our study was that PRP tends to increase faster than stem cells and HA. Our results predict that this increase in PRP will continue until 2026. Although there was a downward trend across all treatments during the COVID-19 period, the number of searches appears to have accelerated since 2022.

The decline observed during the COVID-19 period should be interpreted within a broader healthcare context. During the pandemic, access to elective healthcare services was substantially restricted, and many nonurgent musculoskeletal conditions were deprioritized. In addition, public attention shifted significantly toward COVID-19–related concerns. Therefore, the reduction in search volumes during this period likely reflects not only a temporary decrease in interest, but also a broader disruption in healthcare-seeking behavior. The subsequent increase observed after 2022 may reflect the normalization of healthcare services and a renewed interest in nonsurgical treatment options.

Platelet-rich plasma activates cartilage cells, promotes protein synthesis, and promotes tissue healing within the joints. There is no consensus in the literature regarding the use of PRP in OA, and the results of randomized controlled trials are conflicting.^[11]^ A recent meta-analysis reported that compared with HA and other alternative treatments, PRP significantly reduced OA-related symptoms and pain.^[12]^ Two studies investigating Google Trends data and presenting data up to 2021 reported a growing trend for PRP in the treatment of OA.^[9,12,13]^ Jang et al reported that stem cells were identified more frequently than RSV, HA, and PRP until 2021. Our study suggests that PRP outperforms RSV, HA, and stem cells according to Google Trends until 2025. Based on our ARIMA analysis, we believe this trend will continue to increase.

Degeneration of the articular cartilage and menisci plays a major role in the pathophysiology of OA. Inflammation and fibrosis in the infrapatellar fat tissue and synovial membrane accompany this process. Therefore, reducing inflammation may alleviate the structural deterioration of the knee joint and contribute to OA treatment. Stem cells, with their potential to differentiate into multiple cell types, are used with the theory that they facilitate regeneration despite the destructive process that also occurs in OA treatment. It was recently reported to have reached a market share of $18 billion in the United States.^[14]^ A meta-analysis of 8 randomized controlled trials in 2025 reported that stem cell therapy was superior to placebo in relieving symptoms at 6 and 12 months.^[15]^ Studies investigating Google Trends indicate an increasing RSV volume over the last 10 years until 2019.^[10]^ While it was the most researched regenerative therapy until 2021, our study demonstrated that this trend has remained stable in recent years.

Another alternative to surgery is the intra-articular injection of HA. HA is a naturally occurring, non-sulfated glycosaminoglycan, which is a protein-free compound. Its structure contains repeating units of β-1,4-d-glucuronic acid and β-1,3-*N*-acetylglucosamine.^[16]^ Although clinical evidence is unclear, it is likely that it is gaining increasing interest in routine practice.^[17]^ A study examining the impact of increased interest in the Internet highlighted increased search volumes between 2009 and 2019.^[8]^ Our study results are consistent with the findings of the present study. Although a downward trend, as with all treatments, was observed for COVID-19, an increase in the number of repeat searches was detected starting in 2022.

Studies have shown that OA is associated with season and weather conditions. A meta-analysis of 14 studies reported that KOA is inversely proportional to air temperature and linearly related to humidity and barometric pressure.^[18]^ However, contrary to these data, our study revealed no seasonal differences in search volume. Neither one-way ANOVA comparisons nor time series analysis using the ARIMA forecasting model revealed a statistically significant difference between the seasons. Studies in the literature suggest that search volumes do not differ by season, and results consistent with our study have been reported.^[7-10]^ These findings suggest that public interest in digital OA may be independent of seasonality.

Our study has several limitations. First, variability in search behavior represents an inherent limitation of Google Trends–based analyses. Users may search for the same treatment using different terms, including abbreviations, synonyms, lay expressions, or non-English phrases. Although search terms were selected based on clinical relevance and preliminary testing, this approach may not fully capture the entire spectrum of global search behavior and may introduce selection bias. Second, Google Trends data reflect only users of this specific search engine, and search behaviors may vary across countries and regions. This may have led to geographical bias, particularly in non-English-speaking populations. Third, the absence of demographic data such as age and sex limits the interpretability of the findings. In particular, elderly individuals – who represent a large proportion of patients with KOA – may be underrepresented due to limited access to or familiarity with digital platforms. Fourth, seasonal analyses may be affected by hemispheric differences. While a large proportion of the global population resides in the Northern Hemisphere and experiences similar seasonal patterns, opposite seasonal trends in the Southern Hemisphere may introduce bias into the analysis. Finally, the study focused on 3 commonly used regenerative treatment modalities. Other emerging or alternative therapies were not included, which may have resulted in an underestimation of the overall trends in public interest.

## 5. Conclusion

In conclusion, global online interest in regenerative injection therapies for KOA has increased over the last decade, with the most pronounced rise observed for PRP. HA demonstrated a more moderate upward trend, whereas stem cell–related searches, although initially dominant, have stabilized and declined in recent years. These findings suggest that public interest in regenerative treatment options is dynamic and may evolve independently of clinical evidence. Understanding these trends may help clinicians better anticipate patient expectations and improve patient counseling regarding available treatment options. In addition, these data may assist researchers in identifying emerging areas of interest and aligning future research priorities with evolving public attention. However, it should be emphasized that Google Trends reflects public interest rather than clinical efficacy, and therefore, these findings should not be interpreted as evidence of treatment effectiveness.

## Author contributions

**Conceptualization:** Hakan Uslu, Osman Çiloğlu.

**Data curation:** Hakan Uslu.

**Formal analysis:** Hakan Uslu.

**Investigation:** Hakan Uslu.

**Methodology:** Oğuzhan Çiçek.

**Validation:** Hakan Uslu, Oğuzhan Çiçek.

**Visualization:** Hakan Uslu.

**Writing – original draft:** Hakan Uslu.

**Writing – review & editing:** Hakan Uslu, Oğuzhan Çiçek, Osman Çiloğlu.

## References

[1] Johnson VL Hunter DJ. The epidemiology of osteoarthritis. Best Pract Res Clin Rheumatol. 2014;28:5–15. 24792942 10.1016/j.berh.2014.01.004

[2] Courties A Kouki I Soliman N Mathieu S Sellam J. Osteoarthritis year in review 2024: epidemiology and therapy. Osteoarthritis Cartilage. 2024;32:1397–404. 39103081 10.1016/j.joca.2024.07.014

[3] DeRogatis M Anis HK Sodhi N. Non-operative treatment options for knee osteoarthritis. Ann Transl Med. 2019;7(Suppl 7):S245. 31728369 10.21037/atm.2019.06.68 PMC6828999

[4] Shih M Hootman JM Kruger J Helmick CG. Physical activity in men and women with arthritis national health interview survey, 2002. Am J Prev Med. 2006;30:385–93. 16627126 10.1016/j.amepre.2005.12.005

[5] Sodhi N Piuzzi NS Dalton SE. What influence does the time of year have on postoperative complications following total knee arthroplasty? J Arthroplasty. 2018;33:1908–13. 29352687 10.1016/j.arth.2017.12.020

[6] Zhang W Ouyang H Dass CR Xu J. Current research on pharmacologic and regenerative therapies for osteoarthritis. Bone Res. 2016;4:15040. 26962464 10.1038/boneres.2015.40 PMC4772471

[7] Nuti SV Wayda B Ranasinghe I. The use of google trends in health care research: a systematic review. PLoS One. 2014;9:e109583. 25337815 10.1371/journal.pone.0109583 PMC4215636

[8] Cohen SA Brophy RH Chen AF Roberts KC Quinn RH Shea KG. Public interest in hyaluronic acid injections for knee osteoarthritis in the United States and Europe: an international Google trends analysis. Arthroplast Today. 2022;18:157–62. 36353188 10.1016/j.artd.2022.09.003 PMC9638715

[9] Cohen SA Zhuang T Xiao M Michaud JB Amanatullah DF Kamal RN. Google trends analysis shows increasing public interest in platelet-rich plasma injections for hip and knee osteoarthritis. J Arthroplasty. 2021;36:3616–22. 34172346 10.1016/j.arth.2021.05.040 PMC8478783

[10] Strotman PK Novicoff WM Nelson SJ Browne JA. Increasing public interest in stem cell injections for osteoarthritis of the hip and knee: a Google trends analysis. J Arthroplasty. 2019;34:1053–7. 30935801 10.1016/j.arth.2019.03.002

[11] Blaga FN Nutiu AS Lupsa AO Ghiurau NA Vlad SV Ghitea TC. Exploring platelet-rich plasma therapy for knee osteoarthritis: an in-depth analysis. J Funct Biomater. 2024;15:221. 39194659 10.3390/jfb15080221 PMC11355339

[12] Oeding JF Varady NH Fearington FW. Platelet-rich plasma versus alternative injections for osteoarthritis of the knee: a systematic review and statistical fragility index-based meta-analysis of randomized controlled trials. Am J Sports Med. 2024;52:3147–60. 38420745 10.1177/03635465231224463

[13] Jang CW Bang M Park JH Cho HE. Impact of changes in clinical practice guidelines for intra-articular injection treatments for knee osteoarthritis on public interest and social media. Osteoarthritis Cartilage. 2023;31:793–801. 36813156 10.1016/j.joca.2022.12.013

[14] Ng M Song S Piuzzi NS. Stem cell industry update: 2012 to 2016 reveals accelerated investment, but market capitalization and earnings lag. Cytotherapy. 2017;19:1131–9. 28807603 10.1016/j.jcyt.2017.07.006

[15] Cao M Ou Z Sheng R. Efficacy and safety of mesenchymal stem cells in knee osteoarthritis: a systematic review and meta-analysis of randomized controlled trials. Stem Cell Res Ther. 2025;16:122. 40055739 10.1186/s13287-025-04252-2 PMC11887158

[16] Gupta RC Lall R Srivastava A Sinha A. Hyaluronic acid: molecular mechanisms and therapeutic trajectory. Front Vet Sci. 2019;6:192. 31294035 10.3389/fvets.2019.00192 PMC6603175

[17] Vangsness CT Jr Adamson TC 3rd Daley MJ. Consequences on private insurance coverage: the AAOS clinical practice guidelines and hyaluronic acid injections. J Bone Joint Surg Am. 2020;102:920–6. 32079873 10.2106/JBJS.19.00272 PMC7508284

[18] Wang L Xu Q Chen Y Zhu Z Cao Y. Associations between weather conditions and osteoarthritis pain: a systematic review and meta-analysis. Ann Med. 2023;55:2196439. 37078741 10.1080/07853890.2023.2196439 PMC10120534

